# CD11b is involved in coxsackievirus B3-induced viral myocarditis in mice by inducing Th17 cells

**DOI:** 10.1515/biol-2020-0085

**Published:** 2020-12-31

**Authors:** Heng Wei, Chong-Kai Lin, Sheng-Jian Lu, Yu-Xin Wen, Shuai Yuan, Yan-Li Liu

**Affiliations:** Department of Geriatric Cardiovascular Medicine, First Affiliated Hospital of Guangxi Medical University, No. 6 Shuangyong Road, Nanning City, 530021, China; Graduate School of Guangxi Medical University, No. 22 Shuangyong Road, Nanning City, 530021, China

**Keywords:** CD11b, coxsackievirus B3, viral myocarditis, Th17 cells

## Abstract

Viral myocarditis (VMC) caused by coxsackievirus B3 (CVB3) infection is a life-threatening disease. The cardiac damage during VMC is not mainly due to the direct cytotoxic effect of the virus on cardiomyocytes but mostly involves the induction of immune responses. Integrin CD11b plays an important role in immune response, for instance, in the induction of Th17 cells. However, the role of CD11b in the pathogenesis of VMC remains largely unknown. In the present study, a mouse model of VMC was established by CVB3 infection and CD11b was knocked down in the VMC mice by transfection with siRNA-CD11b. The expression of CD11b and IL-17 in heart tissues, frequency of Th17 cells in spleen tissues and serum IL-17 levels were measured using quantitative RT-PCR, Western blot, immunohistochemistry, flow cytometry and ELISA. Results showed that CVB3 infection caused the pathological changes in heart tissues with the increases in the following indexes: expression of CD11b and IL-17 in heart tissues, frequency of Th17 cells in spleen tissues and serum IL-17 levels. The expression of CD11b was positively correlated with IL-17 expression in heart tissues. Depletion of CD11b attenuated the damage caused by CVB3 and decreased the frequency of Th17 cells in spleen tissues as well as in IL-17, IL-23 and STAT3 expression in heart tissues. In summary, our findings reveal that disruption of CD11b function reduced CVB3-induced myocarditis, suggesting that CD11b may be a novel therapeutic target for VMC.

## 1 Introduction

Viral myocarditis (VMC), which is primarily induced by coxsackievirus B (CVB), is a life-threatening disease. Massive replication of CVB and other viruses in cardiomyocytes triggers the strong host immune response characterized by the infiltration of immune cells and secretion of inflammatory factors, which result in myocardial damage [1,2]. Coxsackievirus B3 (CVB3) is a member of the positive-stranded RNA virus family, Picornaviridae, which has been reported to have recently caused 5–20% of cases of VMC [3,4,5]. Furthermore, CVB3 infection results in irreversible cytopathic effects at the cellular level and cardiac damage at the tissue level [4,6]. Th17 cells are a newly discovered group of CD4^+^ T-assisted cells. Studies have shown that Th17 cells mediate the tissue damage of a variety of autoimmune diseases [7,8]. Previous studies suggest that Th17 cell subsets and their effector IL-17 participate in the pathogenesis of VMC, because of the accumulation of Th17 cells throughout the course of VMC and the role of IL-17 in raising a variety of inflammatory factors, such as TNF-α, IL-1 and IL-6 [9,10,11].

Integrin is the main family of cell surface receptors formed by two subunits of α (120–185 kD) and β (90–110 kD). Integrin family proteins are classified according to the associated β-subunit: the β1 (CD29), β2 (CD18) and β3 (CD61). Members of the β2 (CD11/CD18) integrin family represent the most abundant integrins, which is designated by the different α-subunits such as lymphocyte function-associated antigen 1 (LFA-1; CD11a/CD18), Mac-1 (CD11b/CD18) and gp150/95 (CD11c/CD18). Integrin-mediated immune cell migration and cell-to-cell interactions are critical in immune response. β2 integrin (CD11/CD18) plays an important role in both immune response and inflammation. CD11b is widely expressed in a variety of immune cell subsets, such as dilated myocardial cells (DMCs), monocytes, macrophages, granulocytes and natural killer cells, and involved in many biological processes such as cell activation, cell chemotaxis, cytotoxicity and phagocytosis [12,13,14].

The regulatory effect of CD11b on Th17 cell functions has been reported by a few studies. In a mice model of autoimmune encephalomyelitis, CD11b^+^ myeloid suppressor cells (MDSCs) promoted the differentiation of naive CD4^+^ T-cell precursors to Th17 cells resulting in increased production of IL-17A [15]. This promoting effect of CD11b^+^ MDSCs was dependent on IL-1 receptor on CD4^+^ T cells. Selective depletion of MDSCs using gemcitabine attenuated the severity of autoimmune encephalomyelitis with reduced Th17 cells and inflammatory cytokines (IL-17A and IL-1β) in the lymphoid tissues and spinal cords [15]. However, in some antigen-presenting cells (e.g., dendritic cells, DMCs), CD11b^+^ likely plays an inhibitory role in CD4^+^ T activity and the differentiation to Th17 cells, because using the antibody against CD11b^+^ prevents the inhibition of Th17 differentiation by DMCs, while ligation of CD11b on DMCs constricted Th17 cell expansion within the CD4^+^ T cells [16,17,18]. These results suggest that the regulatory effect of CD11b on Th17 cells is probably related to the cells that express CD11b.

In this study, we aimed to investigate the potential roles of CD11b in CVB3-induced myocarditis. The expression of CD11b and IL-17 in heart tissues, frequency of Th17 cells in spleen tissues and serum IL-17 levels were determined in CVB3-infected mice. Moreover, this study knocked down CD11b in mice to determine whether CD11b was involved in the pathogenesis of CVB3-induced myocarditis.

## 2 Materials and methods

### 2.1 Animal treatments

In this study, 50 male BALB/c mice (aged 6- to 8-week-old) were purchased from the Animal Experiment Center of First Affiliated Hospital of Guangxi Medical University (Guangxi, China). The mice were randomly assigned to three groups as follows: control group (*n* = 20), CVB3 group (*n* = 20) and CVB3 + CD11b knockdown group (*n* = 20). In the last two groups, CVB3 infection was performed as described by Li et al. [19]. Briefly, CVB3 (Nancy strain; ATCC, Manassas, VA, USA) was maintained by passage in HeLa cells (CCL-2; ATCC). The viral titer was determined using a 50% tissue culture infectious dose (TCID_50_) assay on HeLa cell monolayers and calculated by the Reed–Muench method. Mice were infected with an intraperitoneal injection of 0.1 mL of phosphate-buffered saline (PBS; Thermo Fisher Scientific, Waltham, MA, USA) containing 10^3^ TCID_50_ of the virus. In the CVB3 + CD11b knockdown group, mice were injected with siRNA-targeting CD11b (GenePharma, Shanghai, China) and EntransterTM-*in vivo* transfection reagent (Engreen Biosystem Co., Ltd., Beijing, China) from the vena caudalis, according to the method previously described [20], before CVB3 injection. Half the mice in each group underwent the following tests 1 week after the treatments; the remaining mice in each group underwent the following tests 2 weeks after the treatments.


**Ethical approval:** The research related to animal use has been complied with all the relevant national regulations and institutional policies for the care and use of animals and has been approved by the National Institutes of Health’s Code of Ethics the Animal Use [21] and Ethics Committee of First Affiliated Hospital of Guangxi Medical University (Guangxi, China). Mice experiment was carried out in strict accordance with the recommendations of the Guide for the Care and Use of Medical Laboratory Animals (Ministry of Health, P. R. China, 1998).

### 2.2 Histopathology and myocarditis scoring

Heart tissues were collected from the mice 7 days after CVB3 infection. The tissues were fixed in 10% formalin and embedded in paraffin. Sections (5 µm thick) were cut and stained with hematoxylin and eosin (H&E). The extent of the myocardial lesions was quantified and scored for severity as follows: 0 = no inflammation; 1 = 1–5 distinct mononuclear inflammatory foci, with the involvement of 5% or less of the cross-sectional area; 2 = more than 5 distinct mononuclear inflammatory foci or the involvement of over 5% but not over 20% of the cross-sectional area; 3 = diffuse mononuclear inflammation involving over 20% of the area, without necrosis; and 4 = diffuse inflammation with necrosis. The analysis was performed in a double-blinded manner by a trained pathologist [22].

For immunohistochemistry (IHC) staining, the slides were routinely deparaffinized and hydrated. After inactivating endogenous peroxidase in 3% hydrogen peroxide, slides were then retrieved in citric acid buffer (pH 6.0) by microwave for 15 min. Slices were blocked in normal goat serum for 30 min at room temperature and incubated with anti-CD11b antibody (1:1,000; Abcam, Shanghai, China) overnight at 4°C. The slides were then washed with tris buffered saline plus tween (TBST) and incubated with appropriate secondary antibody for 2 h at 37°C. The sections were then washed with TBST and stained by using DAB Detection kit (Solarbio, Beijing, China). Finally, the sections were counterstained with hematoxylin.

### 2.3 Quantitative RT-PCR (qRT-PCR) analysis

Total RNA was extracted from the tissue and cell samples using the Trizol reagent (Invitrogen, Carlsbad, CA, USA). Then 2 µg of the total RNA were reversely transcribed by using the SuperScript RT kit from Invitrogen. qRT-PCR was performed using the ABI PRISM7900 Sequence Detection System (Applied Biosystems, Foster City, CA, USA) with PowerUp™ SYBR^®^ Green Master Mix (Thermo Fisher Scientific). The primer information is given in Table 1. The 2^−ΔΔCt^ method was used to analyze the mRNA expression levels relative to that of the control (GAPDH) mRNA [23].

**Table 1:** Primers used for PCR assay

Gene name	Direction	Sequence (5′–3′)	*T*_m_ (°C)	Amplification size
IL-17	Forward	TCCCACGAAATCCAGGATGC	62	75
	Reverse	GGATGTTCAGGTTGACCATCAC	60.9	
CD11b	Forward	GCCTTGACCTTATGTCATGGG	60.4	185
	Reverse	CCTGTGCTGTAGTCGCACT	61.6	
IL-23	Forward	CTCAGGGACAACAGTCAGTTC	60	119
	Reverse	ACAGGGCTATCAGGGAGCA	62	
STAT-3	Forward	CAGCAGCTTGACACACGGTA	62.4	150
	Reverse	AAACACCAAAGTGGCATGTGA	60.6	
GAPDH	Forward	GGAGCGAGATCCCTCCAAAAT	61.6	197
	Reverse	GGCTGTTGTCATACTTCTCATGG	60.9	

### 2.4 Flow cytometry

The percentage of Th17 cells in CD4^+^ T cells was detected according to the method reported in the previous study [24]. Splenocytes were isolated and suspended in RPMI 1640 containing 10% fetal bovine serum, and red blood cells were lysed by incubation for 3 min in ACK lysis buffer (Tiangen, Shanghai, China). The cells were collected and resuspended at a density of 1.0 × 10^6^ cells per mL. The cells were then stimulated for 4 h with 50 ng phorbol 12-myristate 13-acetate and 1 µg ionomycin per mL (Sigma-Aldrich, St. Louis, MO, USA), and cytokine secretion was blocked with 10 lg/mL brefeldin A (Sigma-Aldrich) at 37°C under 5% CO_2_ in a 24-well culture plate (Corning Costar, Corning, NY, USA) in RPMI 1640 medium supplemented with 100 U penicillin and 100 µg streptomycin per mL and 10% fetal bovine serum. Surface markers were stained with PE-labeled anti-mouse CD4 antibody (Biolegend). After the cells were washed, fixed, and permeabilized according to the manufacturer’s instructions (Biolegend, Beijing, China), they were stained intracellularly with an APC-conjugated anti-mouse IL-17A antibody (eBioscience, San Diego, CA, USA). After incubation at 4°C for 30 min, the samples were washed in staining buffer and measured by flow cytometry on a FACSCalibur cell sorter. The data were analyzed using CellQuest software (BD Biosciences, Franklin Lakes, NJ, USA).

### 2.5 Western blot assay

Western blot was performed as previously described [25]. The heart tissues were homogenized on ice for 30 min in a RIPA buffer (Sigma-Aldrich) supplemented with protease inhibitor. The lysates were heated at 95°C for 5 min and loaded on 10% gels (Bio-Rad, San Diego, CA, USA) for sodium dodecyl sulfate–polyacrylamide gel electrophoresis. After electrophoretic separation, the proteins were transferred onto 0.2 µm nitrocellulose membranes (Amersham, Germany), blocked with 5% nonfat milk (in Tris-buffered saline [TBS] + 0.01% Tween) and incubated overnight at 4°C with the following primary antibodies: anti-CD11b (1:1,000; Abcam) and anti-GAPDH (1:1,000; Abcam). The horseradish peroxidase (HRP)-linked secondary antibodies (1:2,000; Abcam) and ECL kit (WBLUR0100; Millipore Corporation, Billerica, MA, USA) were further used to visualize the blots in the membrane. Proteins were finally visualized by an LAS-4000 mini system (Fujifilm, Japan). The intensity of protein bands was quantified with Quantity one software. The proteins of tissue expression were standardized to GAPDH levels.

### 2.6 ELISA

Serum IL-17A concentration was measured using ELISA (R&D Systems, Beijing, China), according to the manufacturer’s instructions. In brief, plates were coated with the diluted capture antibody (100 mL/well) and incubated overnight at 4°C. The serum samples were added to triplicate wells and incubated at 37°C for 2 h. After the samples were washed, the biotinylated detection antibody was added for 1 h, followed by 100 mL of streptavidin-conjugated HRP. TMB substrate (eBioscience) was added to each well. The absorbance was measured at 450 nm.

### 2.7 Statistical analysis

The software GraphPad 8.0 was used to analyze the data. All data were repeated as an independent experiment three times and presented as mean ± standard deviation(SD). Based on ANOVA, *p* < 0.05 was regarded as significantly different among the experimental groups.

## 3 Results

### 3.1 Increased CD11b expression during CVB3-induced mouse myocarditis

**Figure 1:**
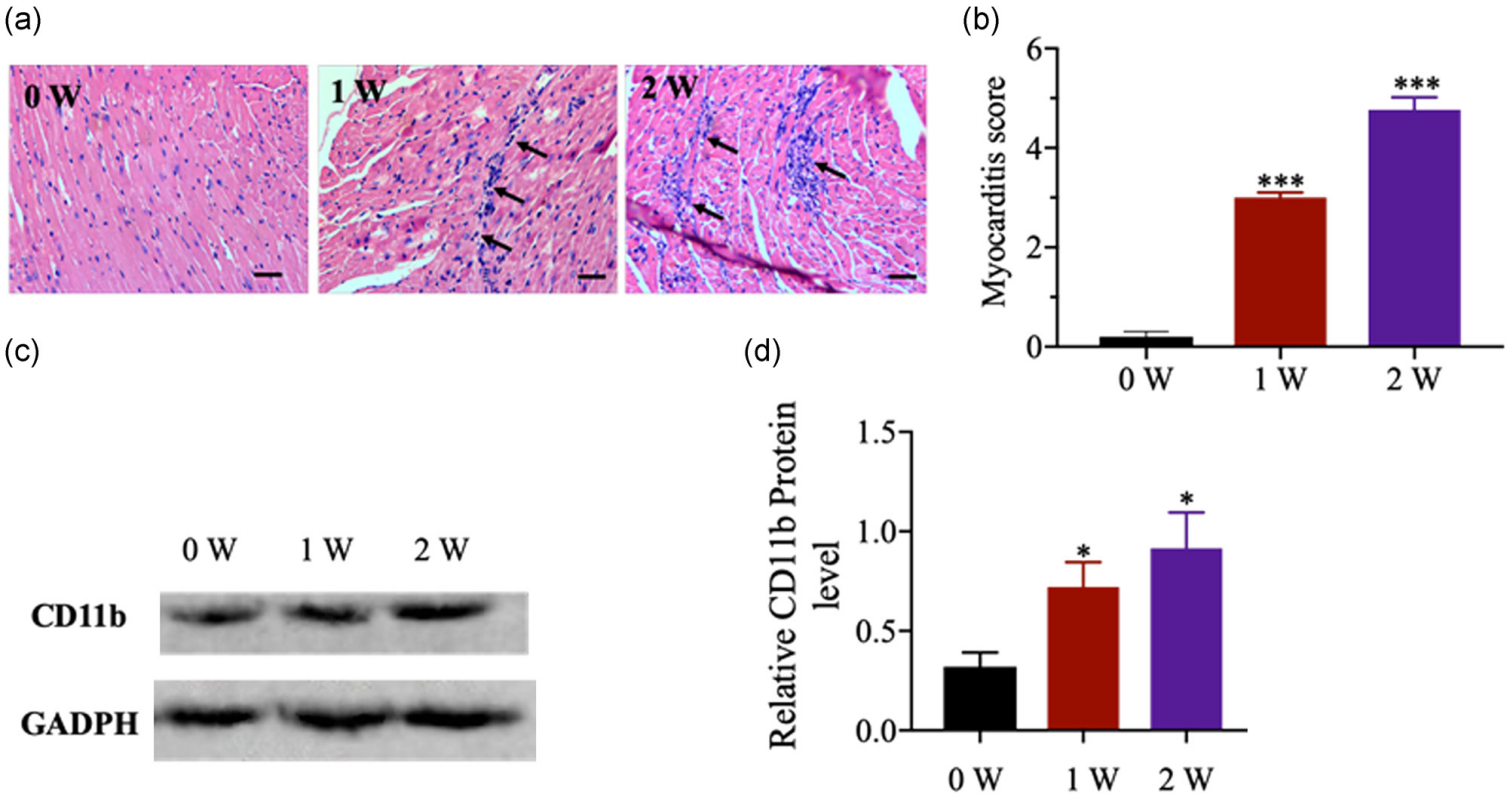
Expression of CD11b was increased during CVB3-induced myocarditis in mice. (a) H&E staining of heart tissue sections was prepared at 1 and 2 week(s) postinfection. Cardiac inflammation was indicated by the arrows (magnification: ×200, scan bar: 50 µm). (b) The degree of the myocardial lesions was quantified and scored according to the method described in previous literature [22]. (c and d) CD11b expression in cardiac tissues at the first and second week(s) after the infection was examined by Western blotting. Data are shown as mean ± SD. *p < 0.05 and ***p < 0.001 compared with the control group.

Male BALB/c mice were injected intraperitoneally with 10^3^ TCID_50_ of CVB3 to construct an acute VMC model. Histological analysis showed higher myocarditis scores in cardiac tissues 1 and 2 week(s) after the CVB3 injection (*p* < 0.001; Figure 1a and b). The expression of CD11b was examined after CVB3 infection. As shown in Figure 1c and d, CD11b increased in the cardiac tissues in the first and second weeks (*p* < 0.05). These results indicated that CD11b expression increased in the cardiac tissues during CVB3-induced mouse myocarditis.

### 3.2 Expression of CD11b and its correlations with the IL-17 expression

**Figure 2:**
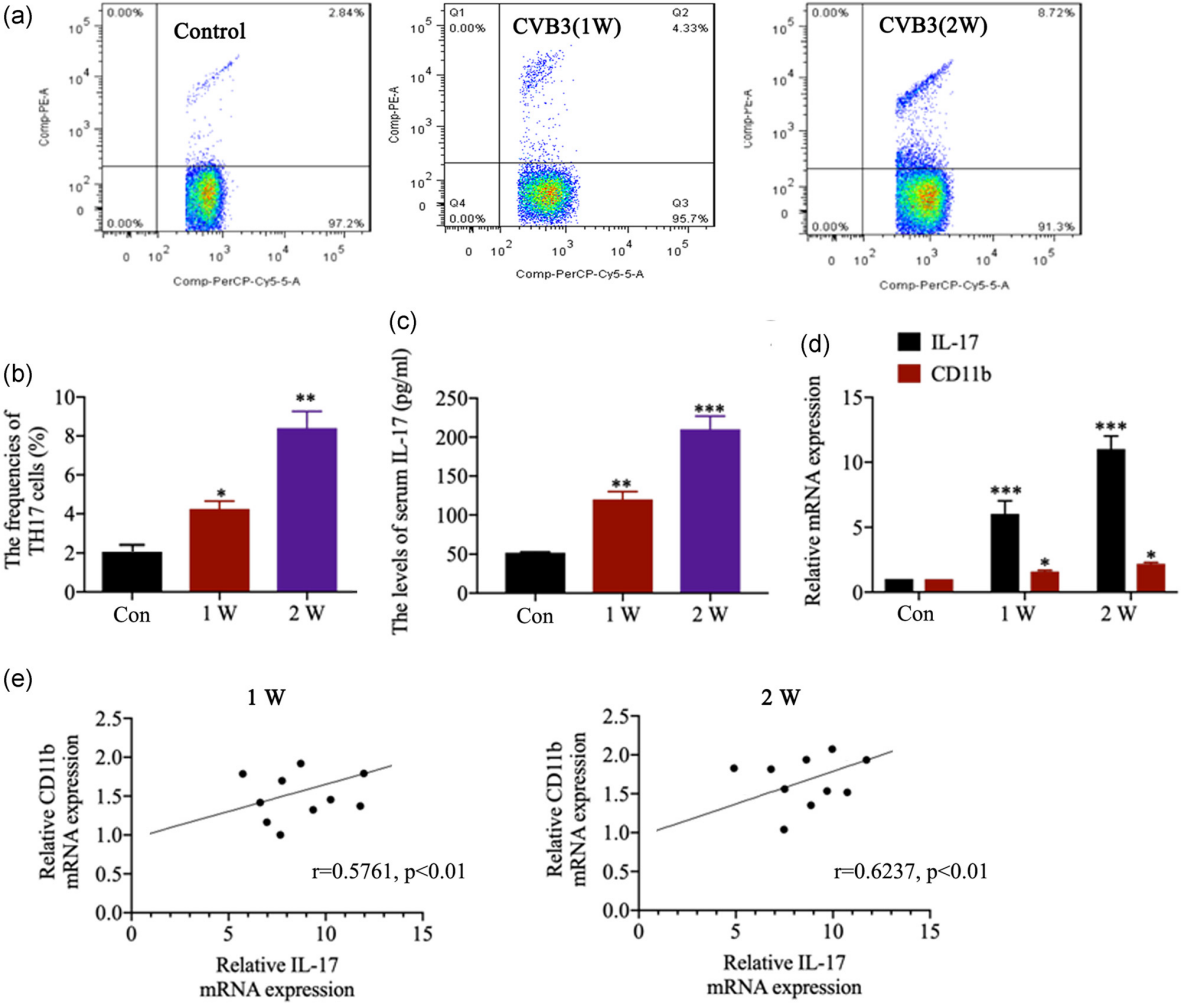
Expression of CD11b and its correlations with the IL-17 expression. (a and b) The frequencies of Th17 cells in CD4^+^ cells in spleen in control and CVB3 mice were analyzed by flow cytometry. (c) The serum IL-17 level in mice was evaluated by ELISA at the first and second week(s) after the infection. (d) The relative IL-17 and CD11b mRNA expression in heart tissues was evaluated by qRT-PCR after CVB3 infection for 1 and 2 weeks. (e) Correlation between CD11b and IL-17 mRNA expression in heart tissues. Data are shown as mean ± SD. *p < 0.05, **p < 0.01 and ***p < 0.001 compared with the control group.

We analyzed the frequencies of Th17 cells in spleen as well as serum levels of the proinflammatory cytokines in CVB3-infected mice. Spleen tissues showed markedly increased frequencies of Th17 cells in mice after infection with CVB3 for 1 (*p* < 0.05) and 2 week(s) (*p* < 0.01; Figure 2a and b). CVB3 infection for 1 and 2 week(s) also caused the elevation of serum IL-17 level (Figure 2c; all *p* < 0.01). The levels of CD11b and IL-17 mRNA were significantly increased in cardiac tissues after infection with CVB3 for 1 and 2 week(s) (Figure 2d; all *p* < 0.05). As shown in Figure 2e, the mRNA of CD11b expression was significantly positively correlated with IL-17 mRNA expression in cardiac tissues after CVB3-infection for 1 (*r* = 0.5761, *p* < 0.01) and 2 week(s) (*r* = 0.6237, *p* < 0.01).

### 3.3 CD11b deficiency ameliorated CVB3-induced myocarditis in mice

**Figure 3:**
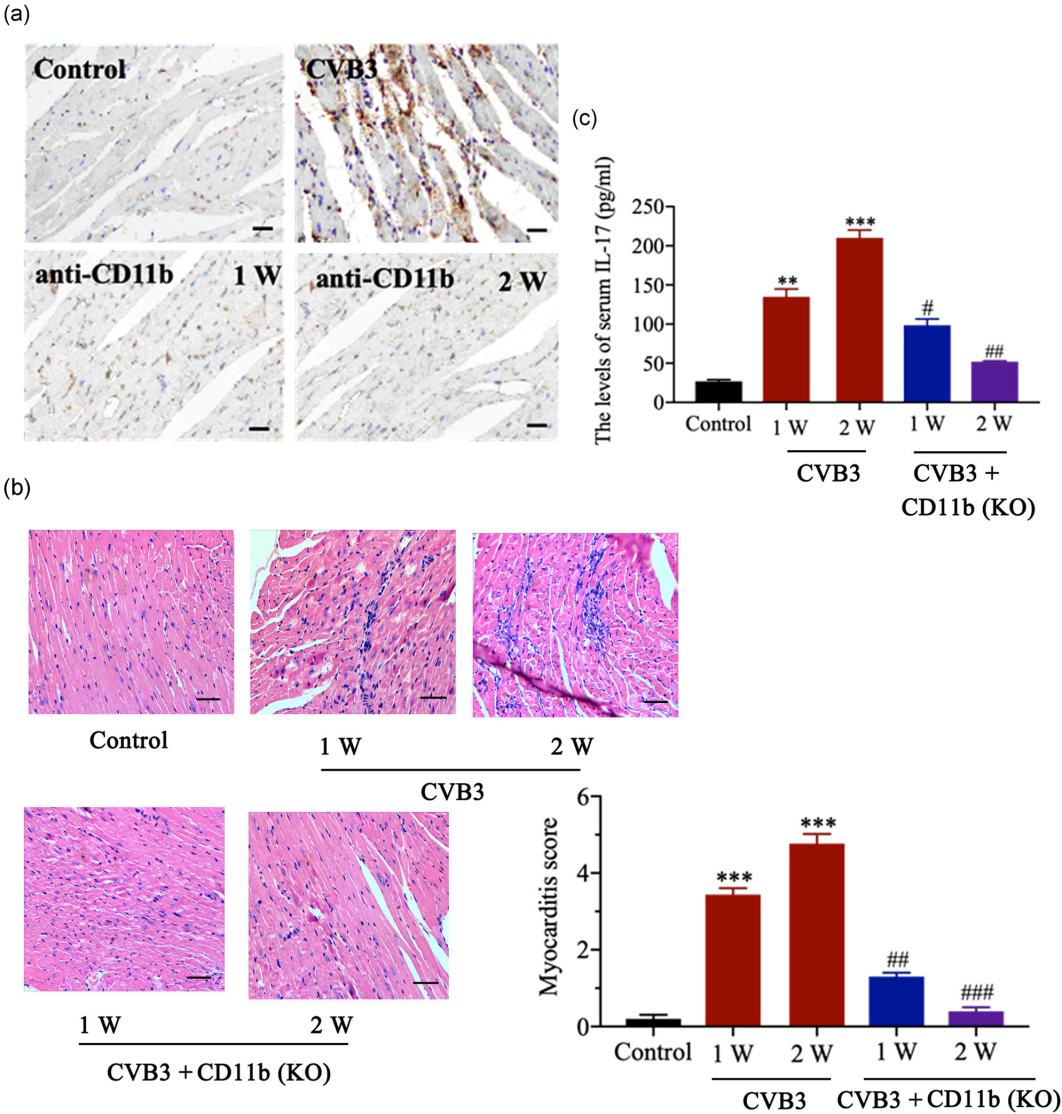
CD11b deficiency ameliorated CVB3-induced myocarditis in mice. Before CVB3 injection, mice were injected with siRNA-targeting CD11b and EntransterTM-*in vivo* transfection reagent from the vena caudalis to knock down CD11b in mice. (a) CD11b expression in heart tissues was assessed using IHC. CVB3: CVB3 injection, anti-CD11b: CD11b knock down. (b) H&E staining of heart tissue sections was prepared at 1 and 2 week(s) postinfection (magnification: ×200, scan bar: 50 µm). The degree of the myocardial lesions was quantified and scored according to the method described in previous literature [22]. (c) The serum IL-17 level in mice was evaluated by ELISA at the first and second week(s) after the infection. Data are shown as mean ± SD. **p < 0.01 and ***p < 0.001 compared with the control group, ^#^ *p* < 0.05, ^##^ *p* < 0.01 and ^###^ *p* < 0.001 compared with the CVB3 group.

To determine the role of CD11b in CVB3-induced myocarditis, this study knocked down CD11b in mice. IHC analysis demonstrated that siRNA-CD11b inhibited the increase in CD11b in heart tissues in CVB3-infected mice (Figure 3a). As indicated by H&E staining, the pathological scores of heart sections in the CD11b-deficient group were lower or less than those in the CVB3 group (*p* < 0.05 in the first week and *p* < 0.01 in the second week; Figure 3b). Although the levels of serum IL-17 in the CVB3 groups increased dramatically (*p* < 0.001), CD11b knockdown decreased the levels of serum IL-17 compared to those in CVB3 groups (*p* < 0.05 or *p* < 0.01). These data indicate that CD11b deficiency ameliorated CVB3-induced myocarditis in mice.

### 3.4 CD11b deficiency suppressed Th17 cell responses

**Figure 4:**
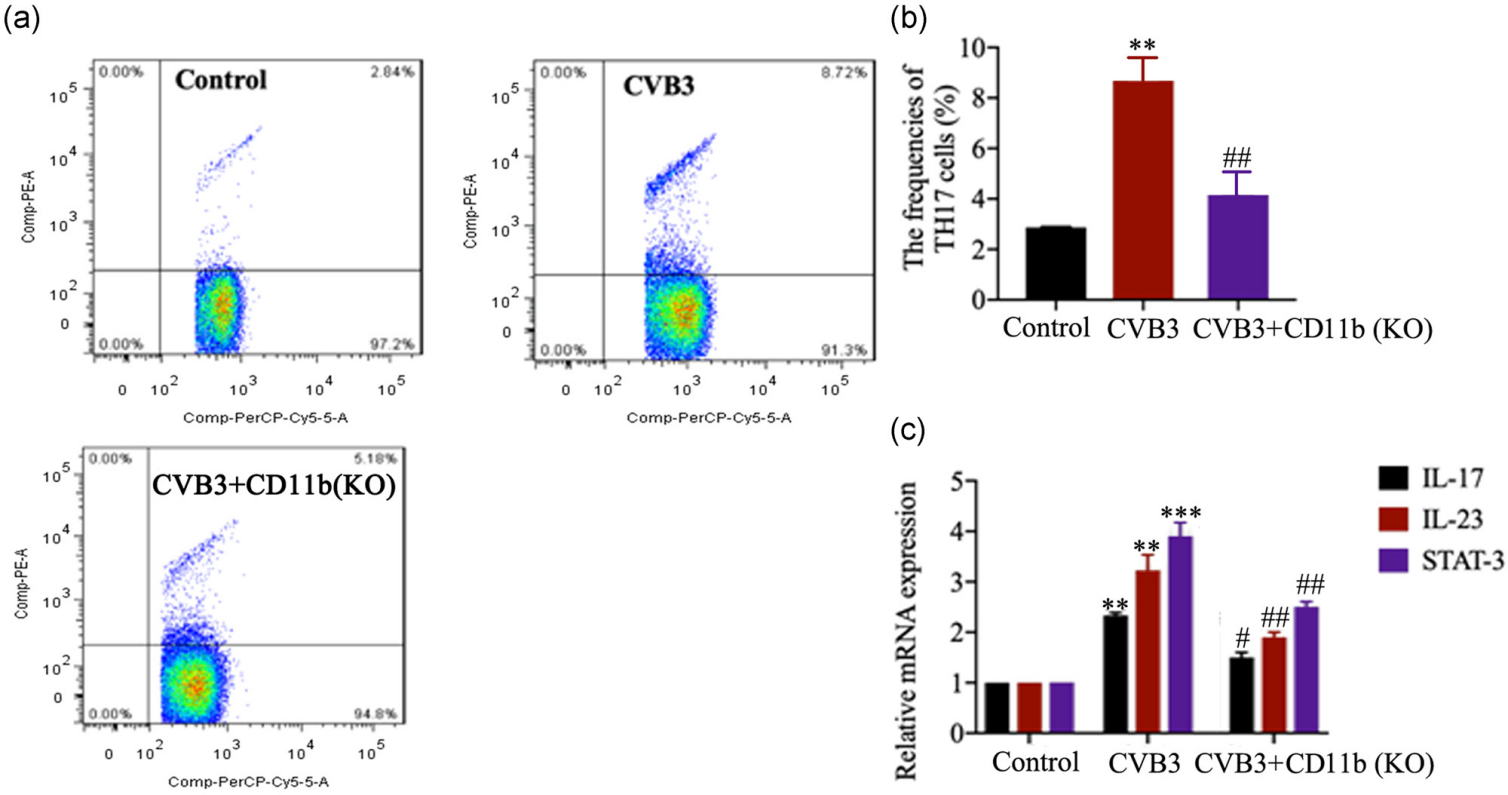
CD11b deficiency suppressed Th17 cell response. Before CVB3 injection, mice were injected with siRNA-targeting CD11b and EntransterTM-*in vivo* transfection reagent from the vena caudalis to knock down CD11b in mice. (a and b) The frequencies of Th17 cells in CD4^+^ cells in spleen in control and CVB3 mice were analyzed by flow cytometry 2 weeks after CVB3 injection. (c) The relative IL-17, IL-23 and STAT-3 mRNA expression in heart tissues was evaluated by qRT-PCR. Data are shown as mean ± SD. **p < 0.01 and ***p < 0.001 compared with the control group, ^#^ *p* < 0.05, ^##^ *p* < 0.01 and ^###^ *p* < 0.001 compared with the CVB3 group.

As shown in Figure 4a and b, CVB3 mice showed markedly increased frequencies of Th17 cells in spleen tissues (*p* < 0.01), while CD11b depletion inhibited the increase in Th17 cells in CVB3 mice (*p* < 0.01). The levels of cardiac mRNA of the IL-17, IL-23 and STAT-3 expression were elevated significantly in CVB3-infected mice as compared to the control mice (*p* < 0.01 or *p* < 0.001; Figure 4c), while the IL-17, IL-23 and STAT-3 mRNA expression markedly reduced in the CD11b-deficient mice compared with the CVB3 mice, especially in the second week (*p* < 0.05 or *p* < 0.01). These data indicate that CD11b deficiency inhibited CVB3-induced Th17 cells and inflammation in mice.

## 4 Discussion

Understanding the pathogenesis of VMC is critical to improve the treatment strategies. The CVB3-infected mouse model summarizes many of the functional and pathological changes in the human VMC disease. In the myocardium of CVB3-infected mice, there is an inflammatory reaction characterized by the infiltration and expression of inflammatory mediators of lymphoid and myeloid cells which are believed to be responsible for the pathogenesis of VMC [26]. The present study showed that CVB3 infection caused the pathological change in the heart tissues of mice with the upregulation of CD11b. The use of siRNA-CD11b to block CD11b function significantly attenuated mouse CVB3-induced myocarditis with the reduction in proinflammatory factors and signaling molecules including IL-17, IL-23 and STAT-3. These data suggested that CD11b may be a therapeutic target for CVB3-induced VMC.

Numerous studies have suggested that cardiac damage during VMC is not mainly due to the direct cytotoxic effect of the virus on cardiomyocytes but mostly involves the induction of immune response [27]. Studies have identified that the differentiation of CD4^+^ cells to Th17 cells plays important role in VMC pathogenesis [9,10,11]. The accumulation of Th17 cells throughout the course of VMC and the release of IL-17 by Th17 cells caused the overproduction of a variety of inflammatory factors, such as TNF-α, IL-1 and IL-6, and consequent inflammatory damage of heart tissues. This study showed that the frequencies of Th17 cells in CD4^+^ cells in spleen tissues were dramatically increased after CVB3 infection. In addition, the serum IL-17 level and cardiac IL-17 mRNA expression levels were increased as well, suggesting the infiltration of spleen-derived Th17 cells to heart tissues. A recent study proposed that VMC pathology is driven by IL-17-producing CD4^+^ T cells because the severity of myocarditis in T-bet-knockout mice was associated with increased IL-17 expression in the heart [28].

Integrin CD11b plays an important role in inducing the adhesion of immune cells to endothelium. Although this study did not provide direct evidence that integrin CD11b facilitated the infiltration of Th17 cells to heart tissues, we found that CD11b expression was positively correlated with IL-17 expression in the heart tissues. Depletion of CD11b also decreased the frequencies of Th17 cells in CD4^+^ cells in spleen tissues, the serum IL-17 level and cardiac IL-17 mRNA expression levels. These data, at least, confirmed that the increase in IL-17 expression in heart tissues is associated with increased integrin CD11b expression. Integrin CD11b not only induced the adhesion of immune cells but also affected the differentiation of CD4^+^ cells to Th17 cells.

The regulatory effect on the differentiation of CD4^+^ cells to Th17 cells is largely dependent on the cells that express integrin CD11b, according to previous studies [15,16,17,18]. CD11b is widely expressed in diverse types of cells, such as myocardial cells, monocytes, macrophages, granulocytes and natural killer cells. However, in this study, it is unclear which type of CD11b-expressing cells drives the differentiation of CD4^+^ cells to Th17 cells. Further study should warrant to clarify this problem. In addition, although this study found increased CD11b expression in the cardiac tissues after CVB3 infection, the underlying molecular mechanism remains unclear. Therefore, an in-depth study on the mechanism is still necessary.

In conclusion, this study found that CD11b expression increased in the cardiac tissues in response to CVB3 infection; CD11b deficiency in turn attenuated CVB3-induced myocarditis in mice via inhibiting Th17 cell and the inflammation responses. Our findings suggest that CD11b might serve as a novel therapeutic treatment of VMC.
